# Post-translational modification-regulated leukocyte adhesion and migration

**DOI:** 10.18632/oncotarget.8135

**Published:** 2016-03-16

**Authors:** Jia Tong Loh, I-hsin Su

**Affiliations:** ^1^ School of Biological Sciences, College of Science, Nanyang Technological University, Republic of Singapore

**Keywords:** adhesion, migration, dendritic cells, EZH2, post-translational modifications

## Abstract

Leukocytes undergo frequent phenotypic changes and rapidly infiltrate peripheral and lymphoid tissues in order to carry out immune responses. The recruitment of circulating leukocytes into inflamed tissues depends on integrin-mediated tethering and rolling of these cells on the vascular endothelium, followed by transmigration into the tissues. This dynamic process of migration requires the coordination of large numbers of cytosolic and transmembrane proteins whose functional activities are typically regulated by post-translational modifications (PTMs). Our recent studies have shown that the lysine methyltransferase, Ezh2, critically regulates integrin signalling and governs the adhesion dynamics of leukocytes *via* direct methylation of talin, a key molecule that controls these processes by linking integrins to the actin cytoskeleton. In this review, we will discuss the various modes of leukocyte migration and examine how PTMs of cytoskeletal/adhesion associated proteins play fundamental roles in the dynamic regulation of leukocyte migration. Furthermore, we will discuss molecular details of the adhesion dynamics controlled by Ezh2-mediated talin methylation and the potential implications of this novel regulatory mechanism for leukocyte migration, immune responses, and pathogenic processes, such as allergic contact dermatitis and tumorigenesis.

## INTRODUCTION

Leukocytes play a central role in our innate and adaptive immunity during both physiological and pathological conditions. As a prerequisite for the elicitation of an effective immune response, these immune cells have to be positioned precisely, in a timely manner, at specific lymphoid and non-lymphoid organs. To achieve this, the leukocyte cytoskeleton is highly flexible and versatile to support rapid and drastic reorganization in response to various stimuli. As such, they are able to generate rapid gliding movements with a migratory velocity of up to 10 μm/min for dendritic cells [1] and 30 μm/min for neutrophils [2], which is almost 100-fold faster than other mesenchymal and epithelial cell types. This gliding motion endows leukocytes with extraordinary migratory capacity that can be described as amoeboid, a migratory pattern reminiscent of the amoeba, *Dictyostelium discoideum* [3]. During amoeboid movement, polarized leukocytes extend their plasma membrane at the leading edge in response to chemoattractants through the polymerization of filamentous actin to form a growing pseudopod. Following attachment of the pseudopod to the underlying substratum, actomyosin contraction at the cell centre transduces an internal force to propel the rigid cell nucleus forward. The trailing edge of the cell then detaches itself from the matrix, facilitating advancement of the cell [4]. Such frequent shape changes experienced by leukocytes during amoeboid migration enables them to squeeze through physically constrained regions of the extracellular matrix, circumventing the need for proteolytic degradation and therefore preserving the structural integrity of the tissue environment [5].

## ADHESION-DEPENDENT AMOEBOID MOVEMENT OF LEUKOCYTES

In order to fulfil their immune functions, leukocytes have to transverse through different tissues composed of distinct extracellular matrices in the body. Depending on the topology of the tissue structures encountered, leukocytes can adopt either an adhesion-dependent or adhesion-independent form of amoeboid movement. When transmigrating across two-dimensional tissue surfaces, such as vessel walls and basement membranes, leukocytes have to rely largely on integrin-mediated adhesion for their effective migration. In this case, the integrin repertoire on the leukocyte surface determines its affinity for the matrix present in the tissue environment. For instance, as circulating blood neutrophils tether and roll along the endothelium, chemoattractant-induced activation of their G-protein-coupled receptors triggers the activation of their surface β2-integrins, αLβ2 and αMβ2, thereby promoting their attachment to the ligand, ICAM-1, expressed on the endothelial cells [6]. As a result of this integrin-mediated adhesion, neutrophils are able to arrest themselves and bind tightly to the endothelial barrier to overcome the shear stress from the flowing blood. This adhesion facilitates the subsequent extravasation of neutrophils from the vascular lumen into the interstitial tissue, hence delivering an effective immune response.

Similar to neutrophils, circulating dendritic cells (DCs) or DC precursors in the blood make use of the integrins, αLβ2 and α4β1, for their firm binding to the endothelial ligands, ICAM-1 and VCAM, respectively. This binding facilitates their trafficking into target tissues during both homeostatic and inflammatory conditions, such as experimental autoimmune encephalomyelitis [7]. On the other hand, Langerhans cells exiting the epidermis have to transverse through a basement membrane network enriched in laminin, type IV collagen and proteoglycans [8] before arriving at the dermal afferent lymphatics for further migration to the skin-draining lymph nodes. In this case, α6 integrin-mediated adhesions have been reported to be critical for attachment and subsequent transmigration across the basement membrane [9].

## ADHESION-INDEPENDENT AMOEBOID MOVEMENT OF LEUKOCYTES

Intriguingly, leukocyte migration in a confined three-dimensional environment was found to be driven primarily by actomyosin contractions, rather than integrin-based adhesions [10]. This was demonstrated by integrin- or talin1-deficient dendritic cells, which, surprisingly, were able to migrate from the dermis to the skin-draining lymph nodes, a route which does not require crossing of any tissue barriers, in a manner indistinguishable from their wild-type counterparts. In this case, the protrusion of actin filaments is sufficient for leukocytes to initiate movement in regions of low matrix density, while myosin II-mediated contraction is only essential when leukocytes have to squeeze through dense regions of tissue interstitium. Indeed, disruption of the actin cytoskeletal network and inhibition of myosin contractile activity through latrunculin B and blebbistatin treatment, respectively, resulted in the migration arrest of leukocytes in a three-dimensional environment [10]. Such an adhesion-independent migration strategy serves to endow leukocytes with the capacity to navigate through different organs rapidly, without being restricted to predefined routes due to the limited integrin repertoire expressed on their cell surfaces.

## DYNAMIC SWITCHING BETWEEN DIFFERENT MODES OF MIGRATION SUPPORTS LEUKOCYTE FUNCTION

Rather than being confined to a particular migratory speed, leukocytes are capable of alternating among fast migration, slow migration and an arrest phase. This dynamic and plastic nature of their locomotion, which depends critically on the shift between adhesion-dependent and -independent migration, is tailored to support distinct functions of the leukocytes. For example, dermal dendritic cells are highly motile cells under steady state conditions, actively crawling through the dermal interstitium in an amoeboid fashion to survey the skin microenvironment for any invading pathogens. However, upon infection with the protozoan parasite *Leishmania major*, they undergo rapid migratory arrest at the infection site to maximise their antigen uptake capacity [11]. Similarly, antigen-specific T cells in the lymph nodes cease migration upon contact with antigen-presenting dendritic cells, forming a stable immunological synapse mediated by αLβ2-ICAM-1 interaction to facilitate full T cell activation [12]. Therefore, striking a balance between cell adhesion and migration in different leukocytes during each of their distinct functional phases plays a key role in modulating a protective immune response.

## POST-TRANSLATIONAL MODIFICATIONS REGULATE CELL MIGRATION

The cell migration machinery is comprised of a myriad of temporally and spatially segregated signalling and adhesion molecules that need to be orchestrated in a rapid and precise manner to support an efficient immune response. To achieve this, post-translational modifications (PTMs) are employed as ideal tools to confer dynamic and reversible regulation in migrating leukocytes. These modifications, which can either act alone or in combination, help to expand the genetic code by increasing the functional diversity of proteins. To date, more than 400 kinds of modifications have been identified [13]. Here, we summarize the major PTMs that have been reported for various cytoskeletal and adhesion-related molecules (Table 1) and highlight their importance with regards to leukocyte migration.

**Table 1:** Selected post-translational modification sites identified on cytoskeletal and adhesion molecules

Molecule	PTM	Site & description
**α-actin**	ADP-ribosylation	Arg-206: Reduces actin polymerization [64]
	Glutathionylation	Cys-374: Reduces actin polymerization [65]
**β-actin**	Arginylation	Asp-3: Promotes actin polymerization [30]
	Methylation	His-73: Stabilization of F-actin [66] Lys-84me1: Disrupts actomyosin interaction [28]
	Sumoylation	Lys-68, Lys-284: Retention of nuclear actin [67]
**γ-actin**	Acetylation	Lys-61: Stabilization of F-actin [68]
**α,β,γ-actin**	ADP-ribosylation	Thr-148: Promotes actin polymerization [69] Arg-177: Promotes actin depolymerization [69]
***Dictyostelium* actin**	Phosphorylation	Tyr-53: Reduces actin polymerization [26]
	Acetylation	Met-1, Asp/Glu-2, Asp-3: Strengthen actomyosin interaction [27]
***Physarum* actin**	Phosphorylation	Thr-201, Thr-202, Thr-203: Promotes actin polymerization [70]
***Drosophila* actin**	Acetylation	Lys-326, Lys-328: Strengthen actomyosin interaction [71]
**Arp2**	Phosphorylation	Thr-237, Thr-238: Promotes actin polymerization [15]
**Cortactin**	Acetylation	Lys-87, Lys-161, Lys-189, Lys-198, Lys-235, Lys-272, Lys-309, Lys-319: Inhibits association with F-actin [72]
	Phosphorylation	Tyr-421, Tyr-466: Promotes Nck 1-dependent actin polymerization [73]
**α-Tubulin**	Detyrosination	C-terminus: Stabilization of microtubules [74]
	Acetylation	Lys-40: Stabilization of microtubules [75]
**β-Tubulin**	Acetylation	Lys-252: Inhibits tubulin incorporation into microtubules [76]
	Phosphorylation	Ser-172: Inhibits tubulin incorporation into microtubules [77]
**γ-Tubulin**	Phosphorylation	Tyr-445: Promotes assembly of astral microtubules [78]
**Non-muscle myosin II light chain A, B, C**	Phosphorylation	Thr-18, Ser-19: Increases association with actin filaments [79] Ser-1, Ser-2, Thr-9: Reduces activity of myosin [80]
**Non-muscle myosin II heavy chain A**	Phosphorylation	Thr-1800, Ser-1803, Ser-1808, Ser-1917, Ser-1943: Promotes disassembly of myosin filaments [79, 81]
**Non-muscle myosin II heavy chain B**	Phosphorylation	Ser-1937: Reduces assembly of myosin filaments [82]
**Non-muscle myosin II heavy chain C**	Phosphorylation	Thr-1932, Thr-1957, Thr-1960: Increases solubility of myosin [83]
**Talin**	Methylation	Lys-2454me3: Promotes disassembly of adhesion structures [50]
	Cleavage*	Gln-433 | Gln-434: Promotes disassembly of adhesion structures [47]
	Phosphorylation	Ser-425: Stabilizes adhesion structures [48]
	Arginylation	Ala-1903: Promotes formation of cadherin-dependent cell-cell adhesion [49]
**Paxillin**	Phosphorylation	Tyr-31, Tyr-118: Promotes docking of SH2 domain proteins [84] Ser-85: Promotes formation of focal adhesions [85] Ser-126, Ser-130: Promotes cell spreading [86] Ser-188, Ser-190: Inhibits paxillin degradation [87] Thr-403: Promotes recruitment of paxillin to adhesion structures [88] Thr-538: Promotes actin depolymerization [89]
	Cleavage*	Ser-96 | Ala-97: Inhibits disassembly of adhesion structures [41]
**Vinculin**	Phosphorylation	Tyr-822: Promotes binding to -catenin for cell stiffening [90]
	ADP-ribosylation	Arg-433: Promotes disassembly of adhesion structures [91]
**Kindlin-2**	Phosphorylation	Tyr-193: Promotes interaction with Migfilin [92]
**Integrin αL**	Phosphorylation	Ser-1140: Promotes high affinity conformation of integrin [93]
**Integrin αM**	Phosphorylation	Ser-1126: Promotes binding to ICAMs [94]
**Integrin αX**	Phosphorylation	Ser-1158: Promotes binding to iC3b and phagocytosis [95]
**Integrin α6**	Cleavage*	Arg-594 | Arg-595: Promotes cell invasion and migration [96]
**Integrin β1**	Phosphorylation	Ser-785: Promotes cell attachment and inhibits cell spreading and migration [97] Tyr-783: Promotes talin binding [98] Tyr-795: Promotes kindlin binding [98]
**Integrin β2**	Phosphorylation	Thr-758: Promotes integrin activation [46] Ser-745, Ser-756: Promotes Dok1 binding [99]
**Integrin β3**	Phosphorylation	Tyr-747: Promotes Dok1 binding [100] Tyr-747, Tyr-759: Inhibits calpain cleavage [45]
	Cleavage*	Tyr-741 | Ala-742, Tyr-747 | Lys-748, Phe-754 | Tyr-755 : Inhibits integrin activation [44] Tyr-759 | Arg-760: Downregulates outside-in signaling [44]
**Integrin β5**	Phosphorylation	Ser-759, Ser-762: Promotes cell migration [101]
**Integrin β4**	Glycosylation	Asn-327, Asn-491, Asn-579, Asn-617, Asn-695: Promotes cell spreading [102]

* Cleavage sites are indicated by a vertical line two amino acid residues.

### Regulation of actin dynamics

The early stage of leukocyte migration initiated by actin filament protrusion at the pseudopod is largely dependent on the interplay between actin polymerization and depolymerization. Such processes are regulated by a family of actin binding proteins including profilin, cofilin, and the actin-related protein (Arp) 2/3 complex. Arp2 is subjected to phosphorylation at Thr-237 and Thr-238 by Nck-interacting kinase (NIK) [14], which stabilizes the Arp2/3 complex in an active conformation required for its actin-nucleating activity [15]. Upon stimulation, activated Arp2/3 initiates nucleation of new actin filaments as branches on the sides of older actin filaments. Once nucleation occurs, profilin-bound ATP actin is incorporated onto the barbed ends of the new actin filaments, resulting in the growth of actin filaments that push the cell membrane forward and promote pseudopod extension. Extracellular stimulation also leads to the release of active cofilin from its inhibitory membrane binding through hydrolysis of PIP2 [16, 17]. Active cofilin at the tip of the leading edge in the proximity of the plasma membrane generates severed actin filaments that are then used as free barbed ends for further actin polymerization [18]. Meanwhile, the activated serine/ threonine kinase, LIM kinase, can also inactivate cofilin through Ser-3 phosphorylation, which probably slows down the rate of filament disassembly behind the leading edge. This inactive cofilin accelerates the dissociation of ADP•G-actin-cofilin and increases the concentration of free ATP•G-actin monomers, making them available for filament elongation at the tip of the leading edge [19, 20]. Interestingly, branches nucleated by Arp2/3 on new actin filaments generated through severing are ten times more stable than those nucleated on old actin filaments [18, 21], which could be the primary mechanism driving the formation of the dendritic actin network at the leading edge. Conversely, during migration arrest or directional change, pseudopods have to be retracted. Under these conditions, the absence of activating signals causes the un-phosphorylated Arp2/3 complex to remain inactive and the type 1 (PP1) and type 2A (PP2A) serine/threonine phosphatases to dephosphorylate and activate cofilin [22], thus increasing contractility at the cell rear through F-actin depolymerization [23].

Apart from the modifications on actin binding proteins, which can indirectly help regulate actin properties, actin itself is also susceptible to many PTMs. Phosphorylation is one of the most prominent modifications found on actin and it is capable of exerting both positive and negative regulatory influences on polymerization. For example, phosphorylation of actin at Thr-201, Thr-202 and Thr-203 by actin-fragmin kinase is known to inactivate the fragmin-actin complex, a complex which promotes the formation of short actin filaments, thereby facilitating the elongation of actin [24, 25]. On the other hand, phosphorylation at Tyr-53 can reduce the rate of nucleation and inhibit the elongation of actin filaments [26]. Thus, different levels of regulation can be imposed through the addition of a single moiety. Moreover, various acetylation sites modified by different acetyltransferases and deacetylases have also been reported on actin. Notably, acetylation of actin at the N-terminus, which has been identified on all kinds of actin, neutralizes a positive charge to strengthen its binding to myosin during the actomyosin ATPase cycle [27], whereas mono-methylation at Lys-84 disrupts the interaction between actin and myosin II [28]. Such interaction plays a critical role in regulating the contractility of the cell, which helps to control migration and other cellular functions. In addition, arginylation of β-actin at Asp-3 by arginyltransferase (Ate1) is known to be necessary for actin polymerization and hence the maintenance of a normal cellular cytoplasmic architecture [29]. In the absence of arginylation, actin filaments undergo aggregation and sequestration at the cell centre, resulting in the collapse of the leading edge, which consequently causes a migration defect in the cell [30]. Taken together, these findings demonstrate the importance of post-translational modifications in the regulation of actin dynamics, which is the key determinant underlying pseudopod formation during cell migration.

### Regulation of actomyosin contraction

Following extension and attachment of the pseudopod to the underlying substrate, actomyosin-mediated contraction of the cell body has to occur to generate forward locomotion. Here, reversible phosphorylation of the motor protein, myosin II, plays an essential role in controlling force generation during migration. Unphosphorylated myosin is inactive and exists in a compact, assembly-incompetent conformation through head-head and head-tail interactions [31]. Upon phosphorylation of Thr-18 and Ser-19 on the regulatory light chain by myosin light chain kinase (MLCK), myosin unfolds to adopt an assembly-competent conformation, thereby increasing its association with actin filaments and also its ATPase activity for the initiation of contraction. This regulation can be counteracted by protein phosphatase 1, which acts to dephosphorylate myosin. In dendritic cells, the distribution of phosphorylated myosin to either the cell front or rear gives rise to fluctuating migratory speeds, which serves to couple their antigen uptake function with their migratory capacity [32, 33]. Several phosphorylation sites have also been identified on the C-terminal region of the myosin heavy chain, including Ser-1917 and Ser-1943. Unlike those found on the regulatory light chain, phosphorylation sites on the heavy chain are associated with the disassembly of myosin filaments. Hence, by making use of phosphorylation switches, myosin is able to modulate its actin-binding and contractile activities to control cell migration.

### Regulation of cell polarization

Following actomyosin contraction, the trailing edge of the leukocyte, termed the uropod, detaches itself from the underlying substratum and undergoes retraction to facilitate forward movement of the cell. Even though contraction of the uropod is dispensable for migration, it has been implicated in promoting shape changes associated with migration through constricted regions of the body. The uropod of motile leukocytes contains the microtubule organizing centre (MTOC) and an extensive network of microtubules, unlike fibroblast-like motile cells, which generally do not form a uropod and place the MTOC between the nucleus and the leading edge [34]. Microtubules, polymers composed of α- and β-tubulin subunits, are uniquely determined by their PTMs, which most likely exert their effects by regulating the binding partners and stability of microtubules. One of the earliest PTMs identified on microtubules was the acetylation of Lys-40 on the β-tubulin subunit. Even though this highly conserved modification is known to be associated with stable and long-lived microtubules, it remains enigmatic how this deeply buried acetyl group within the luminal face of the microtubule can bring about such properties [35]. Interestingly, acetylated microtubules are frequently distributed around the MTOC in the uropod of activated T cells, although their function remains unknown [36]. On the other hand, deacetylation of Lys-40 by histone deacetylase 6 (HDAC6) has been associated with the decreased stability of microtubules and hence an enhanced turnover of adhesion structures [37]. Indeed, disassembly of microtubules that radiate towards the uropod has been reported to enhance the motility of neutrophils [38]. Although many other post-translational modification sites have been mapped out on tubulin, their functional roles in mediating leukocyte migration remain poorly understood.

### Regulation of cell adhesion

During integrin-dependent migration, the protruding pseudopod needs to be stabilized by the formation of new adhesions at the leading edge. Although fast-migratory cell types, like leukocytes, do not usually form visible adhesion structures to mediate their rapid gliding movements, larger adhesion structures may be induced by stimuli to cause the arrest of their migration [39]. Since the dynamic assembly and disassembly of adhesion structures is pivotal to the balance between migration and adhesion, many of these adhesion-related molecules are heavily modified by PTMs. Paxillin is one such focal adhesion-associated adaptor molecule that serves to recruit various cytoskeletal proteins for the transmission of downstream signalling. Phosphorylation of two sites in paxillin (Tyr-31 and Tyr-118), mediated by Src, function to facilitate docking of downstream SH2-containing signalling molecules that subsequently recruit focal adhesion kinase (FAK) to promote the turnover of adhesion structures. Therefore, cells are able to control their adhesion dynamics by regulating the relative proportion of unphosphorylated and phosphorylated paxillin [40]. In addition, paxillin is also susceptible to calpain-mediated proteolysis, which generates an inhibitory paxillin fragment that impairs the turnover of adhesion structures and hence impedes cellular migration [41]. Indeed, macrophages experiencing an increase in calpain-mediated cleavage of paxillin due to a deficiency in CD45, a tyrosine phosphatase, suffer from defects in their cell spreading and migration programmes [42].

Integrins are transmembrane receptors that help to link the extracellular matrix with the actin cytoskeleton. Regulation of these proteins during cell migration and adhesion occurs either through direct post-translational modification or through the PTM of various adaptor proteins such as paxillin, talin, and vinculin. Calpain-mediated cleavage of the integrin cytoplasmic domain has been shown to downregulate outside-in signalling and hence reduce cell adhesion and spreading [43, 44]. This proteolysis, however, can be antagonized by the phosphorylation of the integrin β3 cytoplasmic domain at Tyr-747 and Tyr-759 [45]. Furthermore, the interaction of the integrin β2 cytoplasmic domain with either filamin or talin, which share overlapping binding sites, but possess opposing functions, is modulated through phosphorylation of Thr-758 by protein kinase C (PKC) [46]. This modification favours the binding of talin over filamin, thereby promoting the activation of integrins and their downstream signalling pathways. Such binding of talin to the integrin cytoplasmic tail can, in turn, be antagonized by calpain-mediated proteolysis of talin [47], resulting in the formation of separate head and rod domains. The head domain of talin, which remains integrin-bound, can be ubiquitinated by the E3 ligase, Smurf1, and subsequently degraded, leading to the disassembly of adhesion structures. However, phosphorylation of the head domain by Cdk5 can inhibit the binding of Smurf1 and stabilize focal adhesions [48]. In addition to the head and tail domain, calpain-mediated proteolysis can also generate a 70kDa talin fragment that has been reported to be arginylated on Ala-1903. This modified talin fragment is found to be essential for the formation of cadherin-dependent cell-cell adhesion [49]. More recently, we have identified a talin methylation site on Lys-2454 that plays a critical role in the regulation of leukocyte migration [50]. Methylated talin exhibits reduced binding affinity for F-actin, which promotes the disassembly of adhesion structures and facilitates cellular migration. Collectively, these data demonstrate how adhesion dynamics and cell migration can be intricately regulated by an extensive array of post-translational modifications. In the following paragraphs, we will focus on the enzyme that mediates talin methylation and the functional implications of methylated talin.

## EZH2 REGULATES INTEGRIN-DEPENDENT MIGRATION OF LEUKOCYTES

We have recently reported that Ezh2, a polycomb group protein and well-established chromatin modifier that regulates gene expression through the methylation of lysine 27 on histone H3 (H3K27me), is recruited to talin by Vav1 in the cytoplasm. This recruitment promotes the direct methylation of talin, which alters the binding of talin to filamentous actin and exerts a major influence on cell migration and adhesion dynamics [50]. Our initial observation was that Ezh2-deficient bone marrow-derived mature dendritic cells (DCs) exhibited significantly reduced migratory capacity on integrin ligand coated surfaces compared to control cells. While the fast migrating wild-type mature DCs barely formed any visible adhesions, Ezh2-deficient DCs spread out extensively and frequently formed large and stable adhesion structures with reduced turnover rates. Most of these cells appeared to be trapped on the culture slide and some were observed to be oscillating at an anchored position, as if fighting against the restraining forces created by their binding to the integrin ligands. This migratory defect could be rescued by the expression of wild-type or cytosolic Ezh2, but not an enzymatically inactive Ezh2 or an Ezh2 mutant harbouring mutations that disrupt interactions with the cytoskeletal-reorganization effector, Vav1. Interestingly, when Ezh2-deficient DCs were cultured on surfaces coated with low concentrations of integrin ligands, they formed neither abnormally stable adhesion structures nor spread out extensively. Since the amount of integrin ligand was limited in this case, we believe that the Ezh2-deficient DCs were able to overcome the restraining forces caused by the reduced turnover of adhesion structures and migrate in manners similar to control cells. Taken together, these results demonstrate the integrin-dependent nature of Ezh2-regulated leukocyte migration.

## EZH2-MEDIATED TALIN METHYLATION PROMOTES ADHESION TURNOVER AND CELL MIGRATION

While Ezh2-deficient DCs exhibited clear defects in cell migration, no obvious differences in H3K27me3 levels or gene expression patterns were found between Ezh2-deficient DCs and control cells [50]. However, our whole proteome analysis revealed that various proteins involved in actin cytoskeletal functions were up-regulated (Gunawan et al., unpublished data). Since some of these proteins have the potential to enhance, while others prevent, cell migration or adhesion turnover, we concluded that the phenotype of Ezh2-deficient DCs is therefore unlikely to be caused by their altered expressions. Moreover, we hypothesized that Ezh2-deficiency may affect the function of an integrin proximal molecule, subsequently leading to the formation of stabilized FAs and an accumulation of proteins associated with actin/adhesion structures or the regulation of adhesion dynamics.

**Figure 1:**
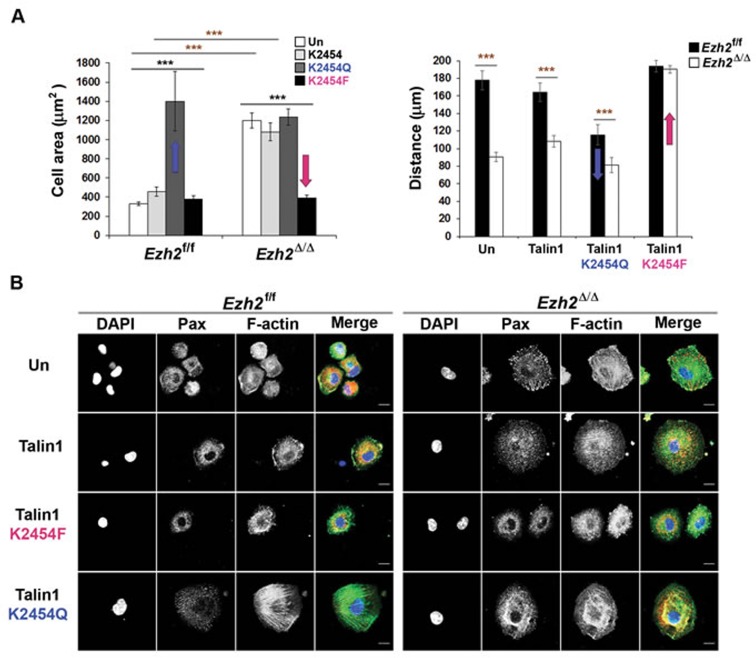
Tri-methyl lysine mimicking talin1 mutant promotes FA turnover and rescues excessive cell spreading and defective migration phenotypes of Ezh2-deficient DCs **A.** Control and Ezh2-deficient DCs expressing GFP-talin1 variants were allowed to adhere to slides coated with VCAM-1 (20 μg/ml) for 2 h. The cell areas were visualized by GFP staining and calculated using ImageJ (left) or time-lapse images were taken every 5 min for 2 h (right). “Un” indicates untransduced control. ***P <0.0001 (black asterisks: one-way ANOVA, red asterisks: between the indicated pairs, two-tailed student's *t*-test with equal variance). Data are represented as mean ± standard error of the mean (SEM) of cells pooled from 2-4 independent experiments. **B.** Control and Ezh2-deficient DCs expressing GFP-talin1 variants were allowed to adhere to VCAM-1 coated slides as in A for 2 h. FAs and F-actin were visualized by anti-paxillin (Pax, red) and Alexa Fluor^®^ 647 phalloidin (pseudo-colored green), respectively. Over 90% of the cells were GFP+ expressing talin variants and the GFP staining is not shown. Scale bar, 10 μm. Technical details and original data are published in reference [50].

Indeed, we found that Ezh2 mediates the tri-methylation of talin1 at lysine (K) 2454 and further demonstrated *in vitro* that such methylation interferes with the binding of talin to F-actin [50]. We further determined that K2454 methylation is critical for the regulation of adhesion dynamics and cell migration by expressing wild-type talin or talin mutants in which the lysine residue was replaced with either an un-methylatable glutamine (Q) residue or with a methyl-mimicking phenylalanine (F) residue (resembles the bulkiness and hydrophobicity of a tri-methylated lysine) in control and Ezh2-deficient DCs. Under all tested conditions, expression of the methyl-mimicking talin1-K2454F mutant restored normal migration and reversed the excessive spreading phenotype of Ezh2-deficient DCs, whereas expression of the un-methylatable talin1-K2454Q mutant converted control cells into Ezh2-deficient DC-like cells (Figure 1A, 1B) [50].

**Figure 2:**
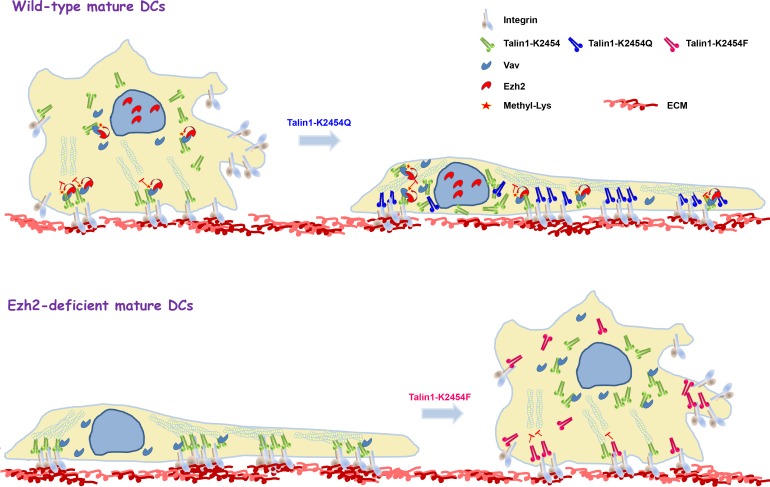
Schematic model for Ezh2-regulated cell adhesion and migration through direct methylation of talin1 Ezh2 is recruited to talin1 through interaction with the cytoskeletal-reorganization effector, Vav1 and mediates talin1 methylation, thereby reducing talin1 binding to F-actin and promoting adhesion turnover. Overexpression in wild-type mature dendritic cells of a mutant talin1 that was created by replacing the lysine at position 2454 with glutamine, which cannot be methylated, but preserves the polarity of the lysine (K2454Q), results in extensive cell spreading that resembles the phenotype of Ezh2-deficient mature dendritic cells (top). In contrast, overexpression in Ezh2-deficient mature dendritic cells of a methyl-mimicking talin1 mutant that was created by replacing the K2454 residue with phenylalanine (K2454F) restores normal cell spreading and migration (bottom).

These results offer convincing support for our hypothesis that Ezh2-mediated talin1 methylation is an important contributing factor for the regulation of adhesion dynamics and cell migration. Furthermore, replacing K2454 with alanine in some of our experiments resulted in trends similar to those seen with glutamine substitution, despite the effects being less pronounced [50]. Since glutamine is not only used to mimic un-methylated lysine with preserved polarity [51], but is also frequently utilized as an acetyl-mimicking residue [52], it would be very interesting to determine whether K2454 may also be subjected to acetylation *in vivo*. In this case, the increased binding affinity of acetylated talin for F-actin may be required for the formation of stable adhesion structures under certain physiological conditions.

## INTEGRIN-DEPENDENT MIGRATION OF CIRCULATING DC PRECURSORS AND LANGERHANS CELLS

Integrin-dependent migration prevails during leukocyte extravasation from the bloodstream into inflamed tissues. However, this process is irrelevant for most DC subsets since they are already residing in the peripheral tissues. In this case, the migration of antigen loaded, active DCs from the periphery to the draining lymph nodes relies on actin polymerization and actomyosin contractions, processes that are independent of Ezh2 function. Accordingly, we recovered similar percentages of FITC-positive migratory dermal DCs in the skin-draining lymph nodes of control and DC-specific Ezh2-deficient mice one day following skin painting with the FITC dye, indicating that integrin-independent migration is intact in Ezh2-deficient DCs [50].

However, unlike other disease models, experimental autoimmune encephalomyelitis (EAE) is one of the exceptional conditions whereby trans-endothelial migration of DCs is critical for disease progression. The extravasation of circulating DCs or DC precursors across the blood-brain barrier into the inflamed tissues of the central nervous system (CNS) requires α4β1 integrin-mediated adhesions [7] and the number of infiltrating DCs has been reported to correlate positively with disease progression [53–55]. In our recent study of EAE, DC-specific Ezh2-deficient mice that exhibited reduced disease scores, compared to control mice, were found to harbor reduced numbers of CNS infiltrating DCs and increased incidences of DCs associated with the microvasculature [50]. These migratory defects observed in Ezh2-deficient DCs *in vivo* are likely to be caused by mechanisms similar to those observed *in vitro*. Furthermore, migration of Langerhans cells (LCs) across the basement membrane requires the binding of integrin α6 expressed on LCs to the integrin ligand, laminin [9], present in the basement membrane layer. Ezh2-deficient LCs, which fail to migrate through the basement membrane under steady state and inflammatory conditions, were found to spread out extensively and form enlarged focal adhesions on laminin coated slides, resembling the phenotype of bone marrow derived DCs [50] (Loh et al., unpublished data). Since LCs have been implicated in the regulation of tolerance induction to skin sensitizers, Ezh2-regulated LC migration may be relevant for the disease progression of allergic contact dermatitis.

## CONCLUSIONS AND FUTURE DIRECTIONS

Integrin-dependent adhesion and migration of leukocytes are critically regulated by post-translational modifications that include phosphorylation, acetylation, sumoylation, ubiquitination and proteolytic cleavage of actin cytoskeletal proteins. These PTMs are frequently amendable by a pair of enzymes that can add or remove modifications on the target proteins. Such dynamic regulation of PTMs enables temporally and spatially controlled signalling cascades and thereby ensures rapid and efficient recruitment of leukocytes in response to invading pathogens.

Recent data published by our lab expands the current role of PTMs in integrin-dependent signalling in leukocytes by suggesting a novel mechanism by which the polycomb group protein, Ezh2, promotes adhesion turnover through direct methylation of the extra nuclear protein, talin1. Interestingly, this Ezh2-mediated talin1 methylation was found to be dependent on its interaction with the cytoskeleton remodelling effector, Vav1 [50]. Subsequent work in our lab has shown that the Ezh2 interaction domain of Vav1 is highly conserved among the Vav family of proteins and that the Ezh2:Vav:talin complexes observed in leukocytes are also formed in non-hematopoietic cells (Venkatesan et al., unpublished). These results suggest that the novel mechanism described here is likely to be conserved across various cell types and may have important implications for tumor growth and metastasis. In fact, Ezh2 overexpression or gain of function mutations are known to be associated with several aggressive human solid tumour types, including prostate cancer, breast cancer, and different types of lymphomas, and are indicators of poor prognosis in patients [56–58]. These Ezh2-dependent oncogenic effects can be achieved though H3K27me3-mediated transcriptional repression of tumor suppressor genes or through promoting NF-κB target gene expression [59–63]. However, our findings suggest that Ezh2-mediated talin methylation may also promote adhesion turnover in cancer cells, resulting in altered adhesion properties that may directly contribute to the epithelial-to-mesenchymal and mesenchymal-to-amoeboid transition of metastasizing cancer cells (Venkatesan et al., unpublished). Furthermore, it is likely that Ezh2 mediates the methylation of additional proteins to modulate other cytosolic signaling events. In addition to talin, several other cytoplasmic proteins were observed to be methylated by Ezh2 *in vitro* (Venkatesan et al., unpublished), so more studies will now be required to determine their identities and physiological relevance.

In conclusion, PTMs play critical roles in regulating cytoskeletal/adhesion dynamics and further research in this area will lead to a better understanding of the regulation of leukocyte trafficking with implications for disease pathogenesis and cellular transformation.
